# Psychosomatic complaints are indicative of stress in young individuals: findings from a Swedish national cohort study

**DOI:** 10.1177/14034948241255179

**Published:** 2024-07-31

**Authors:** Sara Brolin Låftman, Viveca Östberg

**Affiliations:** 1 Department of Public Health Sciences, Centre for Health Equity Studies, Stockholm University, Sweden

**Keywords:** Perceived stress, psychosomatic complaints, health complaints, adolescents, youth

## Abstract

**Aims:**

Psychosomatic complaints are common in youth and are often assumed to indicate stress. Although several studies have confirmed that a cross-sectional association exists, few have empirically investigated whether or not perceived stress influences psychosomatic complaints. The objective of the present study was to build upon previous research by exploring whether changes in perceived stress over time are associated with corresponding changes in psychosomatic complaints. This analytical approach takes unmeasured time-invariant confounding into account, thereby offering more robust evidence for a causal association between the variables under study.

**Methods:**

Data was derived from the Swedish national cohort study Futura01, with information from 2,708 participants aged 17–18 in 2019 and 20–21 in 2022. Perceived stress was measured by Cohen’s Perceived Stress Scale. Psychosomatic complaints were measured by questions on the frequency of stomach aches, headaches and difficulties falling asleep, which were added to an index. Information on gender, parental education, and parental country of birth was derived from registries. Linear regression analyses were conducted and the first difference (FD) approach was used.

**Results:**

Perceived stress and psychosomatic complaints exhibited cross-sectional associations at both time points. The FD analyses showed that increases in perceived stress were associated with increases in psychosomatic complaints, and this was the case irrespective of sociodemographic characteristics.

**Conclusions:**

**This study provides further empirical support for the assumption that psychosomatic complaints can be partially attributed to stress. Societal efforts aimed at reducing stressors and strengthening coping resources and strategies among young people may help mitigate perceived stress and, consequently, the likelihood of developing psychosomatic complaints.**

## Introduction

Psychosomatic complaints, such as headaches, stomach aches, and sleeping difficulties, are prevalent among youth. Studies have indicated an increase in these complaints in several countries [1-3], including Sweden [4-6], in recent years. Despite their common use as an indicator in public health monitoring, the underlying mechanisms of young people’s psychosomatic complaints are not fully clear [7]. While a prevailing assumption suggests that psychosomatic complaints reflect stress, there is limited empirical research on the links between perceived stress and psychosomatic complaints in young people [7]. Existing studies, primarily relying on cross-sectional data, have nonetheless consistently reported positive associations between perceived stress and psychosomatic complaints [7-10].

The objective of the present study was to build upon previous research by exploring whether changes in perceived stress over time are associated with corresponding changes in psychosomatic complaints. A strength of this analytical approach lies in its ability to alleviate potential bias from unmeasured time-invariant confounding, thereby offering more robust evidence for a causal association [11].

## Methods

The data was derived from the Swedish cohort study Futura01, based on a national sample of adolescents attending grade 9 in 2017. At baseline, 5,537 students aged 15–16 completed questionnaires in their classroom (response rate 82%). The second wave was collected through a postal and web survey in 2019, at age 17–18 (*n* = 4,141), and the third wave through a web survey in 2022, at age 20–21 (*n* = 3,396). In the present study, data from the second and third waves were used, as these waves included questions on both perceived stress and psychosomatic complaints. A total of 2,956 individuals participated in both of these waves, and the study sample comprised 2,708 individuals with complete information on the study variables. Additional details about the data material are available elsewhere [12]. In the Supplementary Material, we present a flow chart of the data material (Figure S1) and distributions of the study variables in 2017, 2019, and 2022 (Table S1).

Perceived stress was measured by Cohen’s Perceived Stress Scale with four items (PSS-4) [13]. The portal question read, ‘In the past month, how often have you . . .’ and the items were ‘Felt that you were unable to control the important things in your life?’, ‘Felt confident in your ability to handle your personal problems?’ (reverse), ‘Felt that things were going your way?’ (reverse), and ‘Felt difficulties were piling up so high that you could not overcome them?’. The response categories were ‘Never’, ‘Almost never’, ‘Sometimes’, ‘Fairly often’, and ‘Very often’. The values of the four items were added to an index with the range 4–20 (Cronbach’s α at age 17–18: 0.72; at age 20–21: 0.74). Change in perceived stress was calculated as the difference between the perceived stress score at age 20–21 and at age 17–18. Psychosomatic complaints were measured by questions on the frequency of headaches, stomach aches, and difficulties falling asleep, which were added to an index with the range 3–15 (Cronbach’s α at age 17–18: 0.63; at age 20–21: 0.59). The same items have been used previously to measure psychosomatic complaints [12, 14-16]. Change in psychosomatic complaints was calculated as the difference between the psychosomatic complaints scores at ages 20–21 and 17–18. Information on gender, parental education, and parental country of birth was derived from official registers and linked to the survey data.

Distributions of perceived stress and psychosomatic complaints by sociodemographic characteristics were analysed using *t*-tests and analyses of variance. In an initial step, cross-sectional associations between perceived stress and psychosomatic complaints were examined through linear regression analyses. We also estimated partial eta-squared to assess effect size. For the analyses of changes, the first difference (FD) approach [11] was employed, using change in perceived stress as the independent variable and change in psychosomatic complaints as the dependent variable. To test for differences in the associations by sociodemographic characteristics, interaction terms were included and evaluated by Wald tests. Robust standard errors were estimated, clustering by school class at baseline. All analyses were conducted using Stata, version 17 [17].

## Results

Descriptives of the study sample and mean values of perceived stress and psychosomatic complaints by sociodemographic characteristics are presented in Table I. While perceived stress decreased (in females) between the ages 17–18 and 20–21, psychosomatic complaints did not differ between the two time points.

**Table I.** Descriptives of the study sample and distributions of perceived stress and psychosomatic complaints by sociodemographic characteristics. *P*-values in italics indicate statistically significant differences between groups, assessed with unpaired *t*-tests and analyses of variance (ANOVAs). Bold values indicate statistically significant differences between ages 17–18 and 20–21, assessed with paired *t*-tests (*p* < 0.05).

			Perceived stress age 17–18	Perceived stress age 20–21	Change in perceived stress	Psychosomatic complaints age 17–18	Psychosomatic complaints age 20–21	Change in psychosomatic complaints
	*N*	%	Mean	Mean	Mean	Mean	Mean	Mean
All	2,708	100	**10.66**	**10.30**	–0.36	7.23	7.30	0.06
Gender								
Male	1,127	41.6	9.87	9.82	–0.06	6.25	6.34	0.09
Female	1,581	58.4	**11.22**	**10.64**	–0.59	7.93	7.97	0.04
Unpaired *t*-test			*p* < 0.001	*p* < 0.001	*p* < 0.001	*p* < 0.001	*p* < 0.001	NS
Parental education (highest among parents)								
⩽2 years secondary or less	387	14.3	10.87	10.93	0.06	7.69	7.74	0.05
⩾3 years secondary	503	18.6	**10.81**	**10.32**	–0.48	7.60	7.46	–0.14
Tertiary	1,818	67.1	**10.58**	**10.15**	–0.42	**7.03**	**7.16**	0.12
ANOVA			NS	*p* < 0.001	*p* = 0.013	*p* < 0.001	*p* < 0.001	*NS*
Parental country of birth								
At least one in Sweden	2,305	85.1	**10.62**	**10.21**	–0.41	7.21	7.24	0.03
At least one in Europe	138	5.1	10.77	10.79	0.02	7.33	7.57	0.24
Two parents outside Europe	265	9.8	10.98	10.80	–0.18	7.37	7.61	0.24
ANOVA			*NS*	*p* < 0.001	NS	NS	*p* = 0.048	NS

Linear regression analyses with perceived stress as the independent variable and psychosomatic complaints as the dependent variable demonstrated cross-sectional, positive associations at both time points (Table II). Psychosomatic complaints were more prevalent among females than males, and less prevalent among young individuals whose parents had tertiary education compared with those whose parents had ⩾3 years secondary education. No statistically significant differences were observed based on parental country of birth in the adjusted models. The interaction terms indicated that the associations between perceived stress and psychosomatic complaints differed by gender at both time points, but not by parental education or parental country of birth. The coefficients of the interaction terms and further analyses stratified by gender showed that the associations between perceived stress and psychosomatic complaints were stronger among females than among males (data not presented). Effect sizes in terms of partial eta-squared are reported in the Supplementary Material, Table S2. The results show that perceived stress accounted for about 17% and 18% of the total variance in psychosomatic complaints at ages 17–18 and 20–21, respectively, while controlling for gender, parental education, and parental country of birth.

**Table II.** Results from cross-sectional linear regression analyses of psychosomatic complaints by perceived stress at ages 17–18 (upper panel) and 20–21 (lower panel). *N* = 2,708.

	Psychosomatic complaints aged 17–18			
	Unadjusted^a^		Adjusted^b^	
	*b*	95% CI	*b*	95% CI
Perceived stress 17–18 years	0.42***	0.39, 0.45	0.37***	0.34, 0.41
Gender				
Male (ref.)	0.00	-	0.00	-
Female	1.68***	1.49, 1.87	1.16***	0.98, 1.34
Parental education (highest among parents)				
⩽2 years secondary	0.09	–0.27, 0.46	0.04	–0.29, 0.37
⩾3 years secondary (ref.)	0.00	-	0.00	-
Tertiary	–0.57***	–0.84, –0.29	–0.45***	–0.68, –0.22
Parental country of birth				
At least one in Sweden (ref.)	0.00	-	0.00	-
At least one in Europe	0.12	–0.32, 0.57	–0.07	–0.47, 0.33
Two parents outside Europe	0.16	–0.18, 0.49	–0.09	–0.39, 0.22
Tests for interactions^c^				
Perceived stress*Gender			*p* = 0.045	
Perceived stress*Parental education			NS	
Perceived stress*Parental country of birth			NS	
	Psychosomatic complaints aged 20–21			
	Unadjusted^a^		Adjusted^b^	
	*b*	95% CI	*b*	95% CI
Perceived stress 20–21 years	0.41***	0.38, 0.44	0.38***	0.35, 0.41
Gender				
Male (ref.)	0.00	-	0.00	-
Female	1.63***	1.43, 1.83	1.32***	1.13, 1.50
Parental education (highest among parents)				
⩽2 years secondary	0.28	–0.09, 0.65	–0.02	–0.34, 0.31
⩾3 years secondary (ref.)	0.00	-	0.00	-
Tertiary	–0.30*	–0.56, –0.05	–0.20	–0.41, 0.00
Parental country of birth				
At least one in Sweden (ref.)	0.00	-	0.00	-
At least one in Europe	0.33	–0.15, 0.81	0.01	–0.42, 0.44
Two parents outside Europe	0.36*	0.01, 0.72	0.09	–0.24, 0.43
Tests for interactions^c^				
Perceived stress*Gender			*p* = 0.018	
Perceived stress*Parental education			NS	
Perceived stress*Parental country of birth			NS	

a Includes one independent variable at a time.

b Mutually adjusts for perceived stress, gender, parental education, and parental country of birth.

c Tests for interactions added to the adjusted model, one at a time.

*** *p* < 0.001; *p < 0.05.

The FD analyses, displayed in Table III, showed that increases in perceived stress were positively associated with increases in psychosomatic complaints. While the coefficient for parental tertiary education was statistically significant (indicating a difference in change in psychosomatic complaints by parental education; see also Table I), the associations between change in perceived stress and change in psychosomatic complaints did not vary across groups.

**Table III.** Results from linear regression analyses of change in psychosomatic complaints by change in perceived stress between ages 17–18 and 20–21. *N* = 2,708.

	Change in psychosomatic complaints			
	Unadjusted		Adjusted	
	*b*	95% CI	*b*	95% CI
Change in perceived stress	0.19***	0.16, 0.22	0.19***	0.16, 0.22
Gender				
Male (ref.)	0.00	-	0.00	-
Female	–0.05	–0.23, 0.12	0.06	–0.12, 0.23
Parental education (highest among parents)				
⩽2 years secondary	0.19	–0.15, 0.53	0.04	–0.30, 0.38
⩾3 years secondary (ref.)	0.00	-	0.00	-
Tertiary	0.26*	0.04, 0.49	0.25*	0.03, 0.47
Parental country of birth				
At least one in Sweden (ref.)	0.00	-	0.00	-
At least one in Europe	0.21	–0.21, 0.63	0.15	–0.27, 0.57
Two parents outside Europe	0.21	–0.14, 0.55	0.21	–0.14, 0.56
Tests for interactions^c^				
Change in perceived stress*Gender			NS	
Change in perceived stress*Parental education			NS	
Change in perceived stress*Parental country of birth			NS	

a Includes one independent variable at a time.

b Mutually adjusts for perceived stress, gender, parental education, and parental country of birth.

c Tests for interactions added to the adjusted model, one at a time.

*** *p* < 0.001; *p < 0.05.

## Discussion

This study aimed to investigate the relationship between perceived stress and psychosomatic complaints in a cohort of young individuals in Sweden, with a specific focus on examining whether changes in perceived stress are associated with corresponding changes in psychosomatic complaints. In the initial step, positive, cross-sectional associations between perceived stress and psychosomatic complaints were observed at both ages 17–18 and 20–21, aligning with previous research [7-10]. Approximately 17% to 18% of the variation in psychosomatic complaints could be attributed to perceived stress. These associations were present in both genders, but more pronounced among females than males. The FD analyses revealed that increases in perceived stress over time were accompanied by corresponding increases in psychosomatic complaints, and vice versa, providing further empirical support for the interconnected nature of these two phenomena. The association between changes in perceived stress and changes in psychosomatic complaints did not vary in strength across groups. In summary, the results suggest a positive relationship between perceived stress and psychosomatic complaints that remained irrespective of sociodemographic characteristics. This finding substantiates the notion that psychosomatic complaints can be interpreted as indicators of stress in young individuals [7]. Nonetheless, the results indicated that factors beyond perceived stress also played a role in the occurrence of the types of complaints under study. These factors may encompass medical conditions and hormonal influences as well as social determinants such as social relations and lifestyle factors.

The main strength of this study lies in the prospective data material based on a national sample, encompassing information on perceived stress and psychosomatic complaints at two points in time. This allowed us to employ the FD method, which proves beneficial in addressing omitted variable bias as it accounts for time-invariant confounding [11]. However, it is important to underscore that this approach does not fully address the complexity of causality. Importantly, the method does not account for the directional relationship between the independent and dependent variables. While perceived stress may indeed precede psychosomatic complaints, it is also plausible that these complaints can induce stress. Furthermore, we cannot entirely dismiss the potential influence of a third underlying factor that may contribute to both stress levels and the occurrence of psychosomatic complaints. One limitation of this study was the attrition observed across waves. Although psychosomatic complaints at baseline did not affect the likelihood of participating in subsequent waves [12], it has been noted that males and young people with parents of lower education or foreign-born parents were more likely to drop out [18]. This systematic bias may have compromised the generalisability of the findings. Another drawback is that the measure of psychosomatic complaints relied on only three items with limited internal consistency. The measure predominantly encompasses a somatic component, with two items capturing physical complaints (headaches and stomach aches), while one item reflects psychological complaints (difficulties falling asleep). Notably, Corell et al. found that perceived stress was more strongly correlated with psychological than with somatic complaints [7]. Hence, future studies should examine the links between changes in perceived stress and changes in psychosomatic complaints using scales of somatic and psychological complaints, respectively (cf. [7]).

The data used for this study was collected in 2019 and 2022, encompassing the period coinciding with the COVID-19 pandemic. The majority of participants completed upper secondary school in spring 2020, amidst the pandemic. Studies have suggested that the pandemic may have adversely affected the mental health of young individuals globally [19]. However, a recent Swedish study conducted by Björkegren et al. [20], which focused on remote instruction during the pandemic and its impact on student mental health, showed a decline in healthcare contacts among upper secondary students during and post-pandemic. The result was driven by students in academic programmes. The authors interpreted this finding as potentially indicating unexpected benefits for mental health resulting from remote instruction [20]. Considering the outcomes reported by Björkegren et al., our findings showing a minor decrease in perceived stress (in girls) between ages 17–18 and 20–21, as well as the absence of any difference in the level of psychosomatic complaints between the two time points (see also [16]), appear plausible. Another interpretation of the slight decrease in perceived stress over time among females suggests that during the ages of 17–18, when most participants were still in upper secondary school, stress levels are to a large extent influenced by school performance-related factors [21]. Such stressors are also known to have a significant impact on girls [22, 23].

In conclusion, this study adds to the existing empirical evidence supporting the assumption that psychosomatic complaints can be partially attributed to stress. Efforts to reduce more severe and chronic stressors in young individuals’ lives, and efforts to strengthen their coping resources and strategies that serve protectively against stress, may reduce the likelihood of experiencing perceived stress and, in turn, psychosomatic complaints.

## Supplemental Material

sj-docx-1-sjp-10.1177_14034948241255179 – Supplemental material for Psychosomatic complaints are indicative of stress in young individuals: findings from a Swedish national cohort study Supplemental material, sj-docx-1-sjp-10.1177_14034948241255179 for Psychosomatic complaints are indicative of stress in young individuals: findings from a Swedish national cohort study by Sara Brolin Låftman and Viveca Östberg in Scandinavian Journal of Public Health
