# Supplementary material for: Psychosomatic complaints are indicative of stress in young individuals: findings from a Swedish national cohort study
Source: Scand J Public Health. 2024 Jul 31;53(8):939–44. doi: 10.1177/14034948241255179 (PMC12619837; doi:10.1177/14034948241255179)
Supplement: sj-docx-1-sjp-10.1177_14034948241255179 – Supplemental material for Psychosomatic complaints are indicative of stress in young individuals: findings from a Swedish national cohort study [file sj-docx-1-sjp-10.1177_14034948241255179.docx]

School sample, wave 1, 2017:

n_schools_=500

n_students_=2,956^d^

Schools that agreed

to participate, wave 1, 2017:

nschools=343 n_students_=6,769^a^

Students that agreed to participate, wave 1, 2017:

n_students_=5,722^b^

**Fig. SI** Flow chart of Futura01, waves 1-3.

^a^ Present at school on the day of the classroom survey.

^b^ Responded to the classroom survey.

^c^ Responded to the web survey (83%) or the postal survey (17%).

^d^ Participated in all three surveys: waves 1-3.

^e^ Participated in waves 1 and 3, but not in wave 2.

Study population, age 20-21, wave 3, 2022:

n_students_=3,396

Study population, age 17-18, wave 2, 2019:

n_students_=4,141^c^

n_students_=440^e^

Study population, age 15-16, wave 1, 2017:

n_students_=5,537

Table SI. Distributions of the study variables in the full samples from 2017, 2019, and 2022.

|  | 2017 | | 2019 | | 2022 | |
| --- | --- | --- | --- | --- | --- | --- |
|  | (n=5,358-5,537) | | (n=3,969-4,141) | | (n=2,883-3,396) | |
|  | n | % | n | % | n | % |
|  |  |  |  |  |  |  |
| Gender^a^ |  |  |  |  |  |  |
| Male | 2,743 | 49.5 | 1,888 | 45.6 | 1,484 | 43.7 |
| Female | 2,794 | 50.5 | 2,253 | 54.4 | 1,912 | 56.3 |
|  |  |  |  |  |  |  |
| Parental education (highest among parents)^a^ |  |  |  |  |  |  |
| ≤2 years secondary or less | 993 | 18.4 | 660 | 16.2 | 528 | 15.8 |
| ≥3 years secondary | 1,159 | 21.4 | 825 | 20.2 | 646 | 19.3 |
| Tertiary | 3,255 | 60.2 | 2,594 | 63.6 | 2,171 | 64.9 |
|  |  |  |  |  |  |  |
| Parental country of birth^a^ |  |  |  |  |  |  |
| At least one in Sweden | 4,400 | 82.1 | 3,396 | 83.9 | 2,770 | 83.4 |
| At least one in Europe | 291 | 5.4 | 205 | 5.1 | 171 | 5.2 |
| Two parents outside Europe | 667 | 12.5 | 445 | 11.0 | 379 | 11.4 |
|  |  |  |  |  |  |  |
|  |  |  | Mean | s.d. | Mean | s.d. |
|  |  |  |  |  |  |  |
| Perceived stress age 17-18^b^ |  |  | 10.65 | 2.84 | 10.68 | 2.88 |
|  |  |  |  |  |  |  |
| Psychosomatic complaints age 17-18^b^ |  |  | 7.25 | 2.73 | 7.25 | 2.71 |
|  |  |  |  |  |  |  |
| Perceived stress age 20-21^c^ |  |  |  |  | 10.30 | 2.87 |
|  |  |  |  |  |  |  |
| Psychosomatic complaints age 20-21^c^ |  |  |  |  | 7.30 | 2.69 |

^a^ Measured from register information obtained in 2017.

^b^ Measured from survey data collected in 2019.

^c^ Measured from survey data collected in 2022.

Table SII. Partial eta^2^ from fully adjusted linear regression models. n=2,708

|  | Psychosomatic complaints 17-18 years |
| --- | --- |
|  | Partial eta^2^ |
| Perceived stress 17-18 years | 0.17 |
| Gender | 0.05 |
| Parental education (highest among parents) | 0.01 |
| Parental country of birth | 0.00 |
|  |  |
|  | Psychosomatic complaints 20-21 years |
|  | Partial eta^2^ |
| Perceived stress 20-21 years | 0.18 |
| Gender | 0.07 |
| Parental education (highest among parents) | 0.00 |
| Parental country of birth | 0.00 |
